# Supplementary material for: Main Challenges of Incorporating Environmental Impacts in the Economic Evaluation of Health Technology Assessment: A Scoping Review
Source: Int J Environ Res Public Health. 2023 Mar 11;20(6):4949. doi: 10.3390/ijerph20064949 (PMC10049058; doi:10.3390/ijerph20064949)
Supplement: Supplementary file 1 [file ijerph-20-04949-s001.zip › TableS1_LiteratureSearch_v03.pdf]

**Table S1.** Search strategy in Medline–Ovid SP.

| # | Searches | Results |
|---|---|---|
| 1 | Greenhouse Gases/ | 1482 |
| 2 | Carbon/ and emission.ti,ab. | 3055 |
| 3 | (greenhouse gas and (emissions or accounting)).ti,ab. | 6416 |
| 4 | exp Carbon Dioxide/ and emission*.ti,ab. | 5465 |
| 5 | Climate Change/ and mitigation.ti,ab. | 1261 |
| 6 | 1 or 2 or 3 or 4 or 5 | 14,555 |
| 7 | (carbon adj3 cost*).ti,ab. | 1065 |
| 8 | (health technology assessment or HTA or HTAs).ti,ab,kf. | 7462 |
| 9 | 7 or 8 | 8525 |
| 10 | 6 and 9 | 126 |
| 11 | ((health technology assessment or HTA or HTAs) and environmental*).ti,ab,kf. | 70 |
| 12 | ((health technology assessment or HTA or HTAs) adj7 environment).ti,ab,kf. | 25 |
| 13 | 10 or 11 or 12 | 219 |

ti, terms in the title; ab, terms in the abstract; adj***n*** = terms within ***n*** words of each other (any order); kf, terms in keywords provided by author
