# Supplementary material for: Main Challenges of Incorporating Environmental Impacts in the Economic Evaluation of Health Technology Assessment: A Scoping Review
Source: Int J Environ Res Public Health. 2023 Mar 11;20(6):4949. doi: 10.3390/ijerph20064949 (PMC10049058; doi:10.3390/ijerph20064949)
Supplement: Supplementary file 1 [file ijerph-20-04949-s001.zip › TableS2_StudyCharacteristicsParameters_v04.pdf]

Table S2. Main features of the included studies that develop indicators, parameters and data sources for environmental impact inclusion in HTA.

First author (year) [ref]	Aim	Evaluation scope (Country)	Approach: Indicators/ Parameters	Environmental impact quantification	Keywords
Gell (2010) [6]	To show how a health care enterprise and whole supply chains may flip synchronously into a low-carbon evolutionary pathway	Interventions for carbon reduction (UK)	LCA: water, energy, materials, consumables, packaging, services, waste, air emissions	No	carbon, climate change, economy, enterprise, environment, health care
Prassanna (2011) [33]	To determine the potential for energy savings in a radiology department, and potentially the rest of the hospital	Electrical consumption and cost of workstations and monitors in a radiology department (United States)	Non LCA: energy usage	76.31% potential annual energy savings (83,866.6 kWh), equivalent to 11.6 cars, 14.9 barrels of oil or 39 tons of coal	cost savings, energy efficiency, environmental savings, "green" department
Ellis (2013) [23]	To quantify the traveling made by people from one rural area in Australia to access health care and to calculate the associated carbon emissions for planning telehealth services	Telehealth services (Australia)	Non LCA: travel events (CO_2e)	130.87-134.64 tons of CO_2e in 12 months for the 625 participants in the study, representing, in offsets, growing 20 trees for 30 years; and 0.22 tons of CO_2e per capita per year	carbon emissions, environment, health-related travel, population survey, telehealth
Pollard (2013) [24]	To apply a bottom-up approach (the MARKAL model) to the health sector to analyze the broader implications of healthcare policy and service	Secondary healthcare service configurations to achieve a balance between cost savings and carbon reduction (UK)	Bottom-up: healthcare resources, energy, water, travel events (CO_2e)	Secondary healthcare estimated emissions: 5,787 tons of CO_2e. Patient travel adds 2,215 tons of CO_2e	Not reported

	reconfiguration on carbon emissions set against current consumption				
McCarthy (2014) [34]	To evaluate the power consumption of various devices in the radiology department	Power consumption of computers, workstations, air-conditioning and conference equipment in a radiology department (Ireland)	Non LCA: Energy usage (CO_2e)	Annual power consumption and CO_2e emissions: computers (25,040 kWh, 17.7 tons of CO_2e) and workstations (47,490 kWh, 33.5 tons of CO2e), equivalent to over 10 passenger cars; air-conditioning and conference equipment (37,097 kWh, 26.2 tons of CO_2e)	Radiology, informatics, energy saving, greenhouse gas, environment
Richardson (2016) [9]	To measure the nature and quantity of clinical dental waste, and carbon savings of appropriate segregation and recycling	Dental clinical waste (UK)	LCA: waste (CO_2e)	Annual GHG-savings for appropriate segregation and recycling: 0.555 tons of CO_2e	Not reported
Wilkinson* (2019) [9]	To analyze the impact on greenhouse gas emissions of switching metered-dose inhalers (MDIs) for low global warming potential (GWP) inhalers, such as dry powder inhalers (DPIs)	Low global warming potential inhalers (England)	Non LCA: air emissions (CO_2e)	Annual GHG-savings for 50% of inhalers being low GWP devices by 2022: 288,000 tons of CO_2e	Not reported
Thiel (2020) [13]	To describe the development and pilot testing of an audit online tool (Eyefficiency) for cataract surgical services	Cataract surgical services (South Africa, India, UK)	LCA: surgical resources, energy, waste, travel events (CO_2e)	No	carbon, surgery, healthcare, cost, throughput, productivity

Goel (2021) [32]	To identify variability and efficiency opportunities in cataract surgical practices globally using an auditing tool	Cataract surgical services (International)**	LCA: surgical resources, energy, waste, travel events (CO_2e)	Average GHG emissions per cataract extraction ranging 0.040-0.130 tons of CO_2e, equivalent to driving a passenger vehicle between 163 and 522 km	Not reported
McAlister (2021) [11]	To measure the impact of reducing non-urgent pathology testing on pathology collections and associated carbon emissions	Reduction of non-urgent pathology testing (Australia)	LCA: pathology resources (CO_2e)	GHG emissions savings in a 6-month period: 0.132 tons of CO_2e, equivalent to driving an average passenger vehicle 733 km	pathology, medical overuse, sustainable growth, environment, public health
Wilkinson* (2022) [10]	To provide an overview of the healthCARe-Based environmental Cost of Treatment (CARBON) Programme for a broader understanding of the carbon footprint associated with respiratory care	Respiratory care (International)***	LCA: respiratory care resources, carbon footprint of medications	No	asthma; carbon footprint; COPD; greenhouse gas emissions

CO_2e, carbon dioxide equivalent; GHG, greenhouse gas; HTA, health technology assessment; kWh, kilowatt-hour; LCA, Life Cycle Assessment; UK: United Kingdom.

** The authors declare conflicts of interest*

*** Nine sites spread throughout five International Agency for Prevention of Blindness (IAPB) regions*

**** More than 40 countries*
