# Supplementary material for: Main Challenges of Incorporating Environmental Impacts in the Economic Evaluation of Health Technology Assessment: A Scoping Review
Source: Int J Environ Res Public Health. 2023 Mar 11;20(6):4949. doi: 10.3390/ijerph20064949 (PMC10049058; doi:10.3390/ijerph20064949)
Supplement: Supplementary file 1 [file ijerph-20-04949-s001.zip › TableS3_StudyCharacteristicsEE_v04.pdf]

Table S3. Main features of the included studies that develop applied experiences for economic evaluation of environmental impact in HTA.

First author (year) [ref]	Aim	Technology evaluated (Country)	Economic evaluation approach	Environmental impact quantification	Keywords
Smith (2013) [8]	To estimate the carbon footprint of behavioral support services for smoking cessation	Support services for smoking cessation: text-message support, telephone counseling, group counseling and individual counseling (UK)	Two approaches: Carbon-effectiveness: carbon emissions (CO_2e per 1,000 smokers) in terms of two measures of smoking-related health gain: intervention lifetime quitters and QALYs. Cost-effectiveness including carbon emissions: intervention costs plus costs of carbon emissions, per QALY	Carbon emissions per intervention lifetime quitter (tons of CO_2e): text-message support: 0.281; telephone counseling: 0.466; group counseling 0.504; individual counseling: 1.259. Carbon emissions per QALY gained (tons of CO_2e): text message support: 8.143; telephone counseling: 8.619; group counseling: 16.114; individual counseling: 16.372. Total cost per QALY (GBP): text-message support: 564; telephone counseling: 3202; group counseling: 2295; individual counseling: 5651	Not reported
Marsh* (2016b) [25]	To describe a model to estimate the environmental impacts generated by an individual health technology, to illustrate how environmental impacts could be incorporated into HTA, and to describe the	Insulin addition to an oral antidiabetic regimen for patients with type 2 diabetes mellitus (UK)	Non LCA: Adaptation of an existing clinical-economic model to include environmental outcomes, combining direct and indirect costs with carbon intensity data	Treatment and disease management-related carbon emissions per patient (tons of CO_2e): insulin-OAD regimen: 1.686; OAD-only regimen: 0.310. Treatment-of-complications carbon emissions per patient (tons of CO_2e): insulin-OAD regimen: 3.019; OAD-only regimen: 3.337	Health technology assessment, Environmental impacts, Economic evaluation

	challenges this approach is likely to pose				
Ortsäter* (2019) [19]	To perform a budget impact analysis of adopting RESPIMAT re-usable in the Nordics and Benelux that considered both the traditional healthcare costs as well as the environmental impact	Inhaler device (RESPIMAT) re-usable (Denmark, Iceland, Finland, Norway, Sweden, Belgium, Netherlands, Luxembourg)	LCA: Budget impact analysis incorporating traditional healthcare costs as well as costs of carbon emissions estimated using a societal cost per ton of carbon emission.	Annual carbon emissions savings by replacing pressurized metered-dose inhalers with RESPIMAT re-usable: 186 tons of CO_2 (corresponding to a social cost of carbon emissions of €8122) per 1000 replacements, equivalent to the annual CO_2 footprint of 29 EU citizens	Not reported

CO_2e, carbon dioxide equivalent; EU, European Union; GBP, Great Britain Pound; HTA, health technology assessment; LCA, Life Cycle Assessment; OAD, oral antidiabetic; QALY, quality-adjusted life year; UK: United KIngdom.

** The authors declare conflicts of interest*
