# Supplementary material for: Main Challenges of Incorporating Environmental Impacts in the Economic Evaluation of Health Technology Assessment: A Scoping Review
Source: Int J Environ Res Public Health. 2023 Mar 11;20(6):4949. doi: 10.3390/ijerph20064949 (PMC10049058; doi:10.3390/ijerph20064949)
Supplement: Supplementary file 1 [file ijerph-20-04949-s001.zip › TableS4_Figure2-Refs_v02.pdf]

Table S4. List of the particular bibliographic references related to each approach and challenge shown in *Figure 2. Challenges of incorporating environmental impact in the economic evaluation of health technology assessment*.

Area identified	Topic	Reference
Imput data	LCA approach	[11, 12, 16, 19, 21, 28, 35, 52]
	Supply, demands and waste sides	[8, 16, 35]
	Technology's care pathway	[19, 27]
	Management of disease	[12, 27, 33]
	Use of resources	[12, 27, 33]
	Systematic, standardised and validated methods	[12, 14, 16, 21, 34, 35]
	Medical industry involvement: e.g. EPD	[6, 11, 13, 31]
	Specific data collection: burden vs. representativeness	[11, 16, 35]
	Available, accessible, public and update data	[7, 12, 16, 34, 35]
	Development of data research strategies	[20, 31, 32]
EE Methodologies and frameworks	Holistic approaches	[7, 21]
	Mainstream economic model	[7, 34]

	EEIOA, process-based hybrid approaches	[12, 16, 19, 35]
	Efficiency vs. accuracy	[19]
	EE types: “Enriched CUA, CBA, MCDA	[14, 19, 28]
	Scale problem, equity, SDGs	[3, 13, 24, 34]
	Ecological economic model	[7]
	Comprehensive health care EE	[7, 8, 19, 27]
	Broaden the evidence framework	[39, 41, 48, 52]
	Human well-being, equity, long term environmental sustainability	[7, 17, 18, 24]
	Medical industry involvement	[7, 12, 21, 28]
	Medical industry involvement	[7, 54]
	Consensus on preferred SCC methods	[19]
	International environmental agreements	[53]
Decision-making		
	Quality of the process	[34]
	Multidisciplinary approach: health economist and environmental specialist	[34]

Appropriate methodology: e.g. simple decision modifier, MCDA	[14, 19]
Representativeness of input data in EE	[8, 21]

CBA: cost-benefit analysis; EE: economic evaluation; EEIOA: environmentally extended input-output analysis; EI: environmental impact; "Enriched" CUA: cost-utility analysis; EPD: environmental product declaration; HTA: health technology assessment; LCA: life cycle approach; MCDA: multicriteria decision analysis; SCC: social cost of carbon; SDGs: sustainable development goals
